# Pain management in living related adult donor hepatectomy: feasibility of an evidence-based protocol in 100 consecutive donors

**DOI:** 10.1186/s13104-018-3941-1

**Published:** 2018-11-26

**Authors:** Guillaume Dewe, Arnaud Steyaert, Marc De Kock, Fernande Lois, Raymond Reding, Patrice Forget

**Affiliations:** 1 Department of Anesthesiology, Cliniques Universitaires Saint-Luc, Avenue Hippocrate 10, 1200 Brussels, Belgium; 2 Department of Anesthesiology, Centre Hospitalier de Wallonie Picarde, Avenue Delmée 9, 7500 Tournai, Belgium; 3 Department of Anesthesiology, Centre Hospitalier Universitaire du Sart-Tilman, Liège, Belgium; 4 Department of Surgery and Transplantation, Cliniques Universitaires Saint Luc, Avenue Hippocrate 10, 1200 Brussels, Belgium; 5 Department of Anesthesiology and Perioperative Medicine, Vrije Universiteit Brussel (VUB), Universitair Ziekenhuis Brussel (UZ Brussel), Laarbeeklaan 101, 1090 Brussels, Belgium

**Keywords:** Pain management, Living related adult donor hepatectomy

## Abstract

**Objective:**

Living donor hepatectomy (LDH) has important consequences in terms of acute and chronic pain. We proposed an anesthetic protocol based on the best currently available evidence. We report the results of this protocol’s application.

**Results:**

We performed a retrospective descriptive study of 100 consecutive donors undergoing LDH. The protocol included standardized information provided by the anesthetist, pharmacological anxiolysis and preventive analgesia. Specifically, pregabalin premedication (opioid-free) intravenous anesthesia (with clonidine, ketamine, magnesium sulphate and ketorolac) and epidural analgesia were proposed. Postoperative follow-up was conducted by the Postoperative Pain Service. This analysis included 100 patients (53 women, 47 men, median age 32.7 years old [28.4–37.3]), operated by xypho-umbilical laparotomy. All elements of our anesthetic protocol were applied in over 75% of patients, except for the preoperative consultation with a senior anesthesiologist (55%). The median number of applied item was 7 [interquartile range, IQR 5–7]. Median postoperative pain scores were, at rest and at mobilization respectively 3 [IQR 2–4] and 6 [IQR 4.5–7] on day 1; 2 [IQR 1–3] and 5 [IQR 3–6] on day 2; and 2 [IQR 0–3] and 4 [IQR 3–5] on day 3. In conclusion, LDH leads to severe acute pain. Despite the proposal of a multimodal evidence-based protocol, its applicancy was not uniform and the pain scores remained relatively high.

**Electronic supplementary material:**

The online version of this article (10.1186/s13104-018-3941-1) contains supplementary material, which is available to authorized users.

## Introduction

Living donor hepatectomy (LDH) for related pediatric liver transplantation is often associated with significant acute and chronic postoperative pain [1–3]. Pain is undesirable and often underestimated in its multi-factorial dimensions. Consequences of pain and the treatment of pain may be detrimental to postoperative outcome [4] and are potentially preventable [4–6].

In 2012, Bonnet et al. [3] audited postoperative pain after this surgery in our institution. The authors observed, on the 1st day, that 11% and 37% of the patients reported severe postoperative pain (i.e. a score of more than 6 out of 10 on an 11-points numeric rating scale 1), respectively at rest and at movement. Additionally, the patients experiencing persistent pain after surgery presented higher pain scores than the other, highlighting the need for better acute pain management.

Following these results, we aimed to improve the quality of postoperative pain management with an evidence-based protocol. Here, we describe this protocol, systematically assess the level of evidence for each intervention, and report feasibility (i.e. acceptance) in hundred consecutive donors.

## Main text

After ethics committee approval (Number 017/02JAN/003), Chairperson: Jean-Marie Maloteaux), we performed a retrospective study of 100 consecutive donors operated between June 2010 and the February 2014 for LDH. Written informed consent was waived by the ethics committee regarding the retrospective nature of the study. Criteria for inclusion in the liver donation program at our institution are summarized in Table 1. For each donor, we retrieved demographic and surgical characteristics, perioperative anesthetic and analgesic regimen, pain scores, clinical and biological (white blood/red cell counts, platelets, C-reactive protein, liver and renal function, at day + 1 and + 5) follow-up, length of stay, postoperative complications and residual pain 2 months after the surgery.

**Table 1:** Criteria for inclusion for living liver donation

Aged from 21 to 55 years old
Related to the child
BMI ≤ 27
Good overall health (ASA 1 or mild 2)
Negative serology for HVC, HBV, HIV, syphilis and tuberculosis
Absence of anatomical variants which contraindicate the expected resection
Satisfying psycho-social evaluation
Sufficient hepatic parenchyma volume allowing a resection > 5 g/kg of recipient weight
Normal laboratory tests (blood compatibility, glycaemia, urea nitrogen, creatinine, direct and total bilirubin, GGT, cholesterol, blood cells count, INR, αFP)
Normal chest X-ray
Mild (or moderate) hepatic steatosis is tolerated

### Procedures

The perioperative anesthetic and analgesic protocol was based on the most recent and highest level of evidence with the Oxford Center for Evidence-Based Medicine (EBM). The first level of evidence (LoE 1) applies to an intervention approved by meta-analysis. Those approved by Randomized Controlled Trials are considered LoE 2. LoE 3 is for non-randomized controlled trials, LoE 4 for other studies and LoE 5 for mechanism-based reasoning. In the following sections, we detail the LoE for each part of the protocol [7, 8].

#### Preoperative phase

Preoperative information is given by an anesthesiologist (PF, with a translator if needed) and includes a patient-oriented description of the perioperative phases, procedure, (un)anticipated events, pain expectations and treatment, physical and psychological consequences (including on a short and long-term) (LoE 2) [9].

Pregabalin, as an α-2-δ subunit antagonist voltage-gated Ca^2+^ channels, has shown benefits in terms of anxiolysis (LoE 1), chronic pain (LoE 1), reduction of opioid consumption (LoE 1), improving postoperative analgesia (LoE 2), reduction of postoperative hyperalgesia (LoE 2) and prevention of chronic post-surgical pain (LoE 1) [10–13]. Timing of administration is important, as it takes 6 h after oral administration to reach cerebrospinal fluid levels associated with decreased central nervous system sensitization [14]. To reduce preoperative anxiety (which is not limited to the day of surgery) and sleep disturbances [15, 16], and to obtain stable drug concentrations (i.e. after at least 5 half-life, 24–48 h [17, 18]), patients received 150 mg twice a day for 5 days before surgery (LoE 2).

Additionally, on the morning of surgery, as an anxiolytic premedication and to anticipate postoperative pain and emergence agitation, patients receive 150 mcg of oral clonidine (LoE 1) [19].

#### Intraoperative phase

To decrease postoperative pain, analgesic consumption without delaying recovery, intravenous clonidine (an α2-agonist) is added during the induction of general anesthesia, up to 4 mcg/kg [20] (LoE 1). With its unique vasoactive properties, clonidine has the significant advantage of decreasing portal and vena cava pressure, useful in the context of hepatic surgery. It also improves hemodynamic stability and permits opioid-free anesthesia. While adverse effects are well known (hypotension and bradycardia), they have not been associated with any significant sequel, confirming its safety profile [21–23] (LoE 1).

Due to the small number of pediatric liver transplantation centers in the world, most of these donors come from abroad and do not speak either French, English or Dutch. Because of this, and regarding the living donor condition (i.e. typically at risk of high levels of anxiety), and depending on the preference of the patient, we propose an inhalatory induction (sevoflurane), before a large venous catheter placement (LoE 5).

After the induction, the anesthesia is maintained with a continuous infusion of propofol, guided by an electroencephalographic monitoring (Neurowave Systems inc). Propofol lowers the risk of postoperative nausea and vomiting [24, 25] (LoE 1). Moreover, there may be additional arguments for its use in the context of liver transplantation, where inflammation and ischemia–reperfusion injuries are of potential concern. Propofol has shown anti-inflammatory effects [26–30] and may also reduce acute postoperative pain scores [31] (LoE 3).

Opioids are known to cause hyperalgesia [32, 33] and high intraoperative doses are associated with higher postoperative pain level [32] and morphine consumption [34, 35] (LoE 2). Opioid-free anesthesia is a strategy that, when part of a multimodal analgesia regimen [36], suppresses the risk of intraoperative opioid-induced hyperalgesia and decreases the risk of postoperative nausea and vomiting [37] (LoE 2).

Thoracic epidural is the most effective analgesic [38–40] and anti-hyperalgesic technique [40, 41] after major abdominal surgery (LoE 1). It allows opioid-sparing, lowers the incidence of pruritus and improves gut motility (LoE 1). It may decrease the incidence of chronic postoperative pain [40] (LoE 2) and possibly even mortality/morbidity [42–46] (LoE 1). Neuraxial blockade may thus improve recovery [47]. The associated vasodilatation may reduce intraoperative blood losses. Side effects include failure, dural puncture, epidural abscess and hematoma. We systematically propose thoracic epidural analgesia to the donor, after complete information. The epidural catheter is placed using a loss-of-resistance technique, in lateral decubitus position, after the induction of the anesthesia. Levobupivacaine associated to clonidine (respectively a bolus 5 mL 0.5% and 1 mcg/kg is used, followed by a continuous infusion of 5 mL/h of levobupivacaine 0.25%). In case of refusal (or failure) of the epidural, an intraoperative continuous intravenous infusion of lidocaine can be proposed (2 mg/kg/h), associated with a postoperative patient-controlled morphine infusion [48, 49] (LoE 1).

Ketamine is an anti-pro-inflammatory drug [50] and low “anti-hyperalgesic” doses permit opioid-sparing [33, 51–53] (LoE 1), decrease hyperalgesia [32, 53] (LoE 1) and possibly the risk of postoperative chronic pain (LoE 2) [10, 54]. We systematically administer intravenous ketamine (bolus dose of 0.5 mg/kg at induction, followed by an intraoperative infusion of 0.25 mg/kg/h until 30 min before awakening).

Perioperative intravenous infusion of magnesium reduces postoperative pain, morphine consumption and shivering [55, 56] (LoE 1). Our patients routinely receive a loading dose of magnesium sulphate (2–3 g) at the induction of anesthesia.

Administration of ketorolac, a non-steroidal anti-inflammatory drug, reduces postoperative pain and analgesic use [57–61] (LoE 1) and is not associated with an increased bleeding risk [62] (LoE 2). Unless contraindicated, our protocol includes an intraoperative dose ketorolac (0.5 mg/kg, maximum of 30 mg).

A gastric tube, placed after anesthesia induction, is removed before awakening (LoE 1) [63–65].

#### Postoperative phase

All patients are extubated at the end of the surgery and stay 24 h in the intensive care unit.

Our postoperative analgesia regimen includes an epidural (typically levobupivacaine 0.125%, clonidine 0.75–1.5 mcg/mL, sufentanil 0.1 mg/mL; 5 mL/h, bolus 5 mL, lockout 50 min) or intravenous (morphine, bolus 1.4 mg, lockout 7 min, 30 mg max per 4 h for men and bolus 1 mg, lockout 5 min, 25 mg max per 4 h for women) patient-controlled infusion. If necessary, acetaminophen low doses (up to 2 g a day) and ketorolac can be added.

An anesthesiologist and a nurse are dedicated to the Acute Pain Service. An on-call anesthesiologist is available 24/7 [66–69] (LoE 3). In case of ineffective epidural analgesia despite an additional bolus of local anesthetic, the technique is immediately replaced by an intravenous patient-controlled morphine infusion. Pain intensity is evaluated daily with an 11-point-numeric-scale (0–10) and prospectively registered in an electronic database. Pain scores superior or equal to 4 and 7 are considered to be respectively in moderate-to-severe pain group [70] and severe pain group [71].

#### Statistical analyses

Data were presented as numbers (percentage) or median [interquartile—IQR 25–75]. As distributions are not normal (Shapiro–Wilk’s test), statistical comparisons were made with Wilcoxon test followed by Bonferroni’s correction. JMP Pro 12.0.1 statistical package (SAS Institute Inc., Cary, NC, USA) was used for all the analyses.

### Results

#### Donors

This analysis included 100 consecutive donors (53 women, 47 men). The median age was 32.7 years [IQR 28.4–37.3]. Sixty-nine percent of the donors were classified as ASA 1 and 31% as ASA 2. One donor’s anesthesia protocol was missing and excluded from the analyses. For 39 others, postoperative pain scores were incomplete. Available data was considered for analysis.

Seventy percent of the donors were coming from other countries than Belgium (most of them come from Eastern Europe) and did not speak French, Flemish nor English. Nineteen were native from Belgium, 7 from the Netherlands, 1 from Luxembourg and 3 were coming from other countries but living in Belgium. For the others, the postoperative data were considered up to their departure from Belgium.

#### Procedure

In all patients, a midline abdominal incision was followed by the following liver resections: a left liver lobectomy for 90 donors, a left hepatectomy for 6, a full left hepatectomy for 3 and a right hepatectomy for 1 donor. The median length of surgery was 312 min [IQR 279–328]. The different components of the protocol for prevention of pain and hyperalgesia and their rate of applications are summarized in Table 2. Nineteen donors received the entire protocol. Six patients refused the epidural but received all other items. Thirty donors had one element missing and 51 had more than one element missing. Focusing on the epidural analgesia, 8 donors refused the technique; for one other, the insertion of the epidural catheter was unsuccessful.

**Table 2:** Anesthetic and analgesic protocol, level of evidence (LoE) according to the Oxford Center for Evidence Based Medicine, and application rate in a series of hundred consecutive living related adult donor hepatectomy

	LoE	Application rate (%)
Preanesthesia consultation with dedicated anesthetist	1	55
Premedication with pregabalin	1–2	75
Total intravenous anesthesia		83
Thoracic epidural analgesia	1–2	77
Opioid-free anesthesia	OIH → 1	79
Clonidine		
IV	2	87
(Epidural)		91
Ketamine		
Induction	2	93
Continuous infusion		83
Ketorolac	2	78
Magnesium sulphate	1	79

*OIH* opioid induced hyperalgesia

#### Postoperative period

**Fig. 1:**
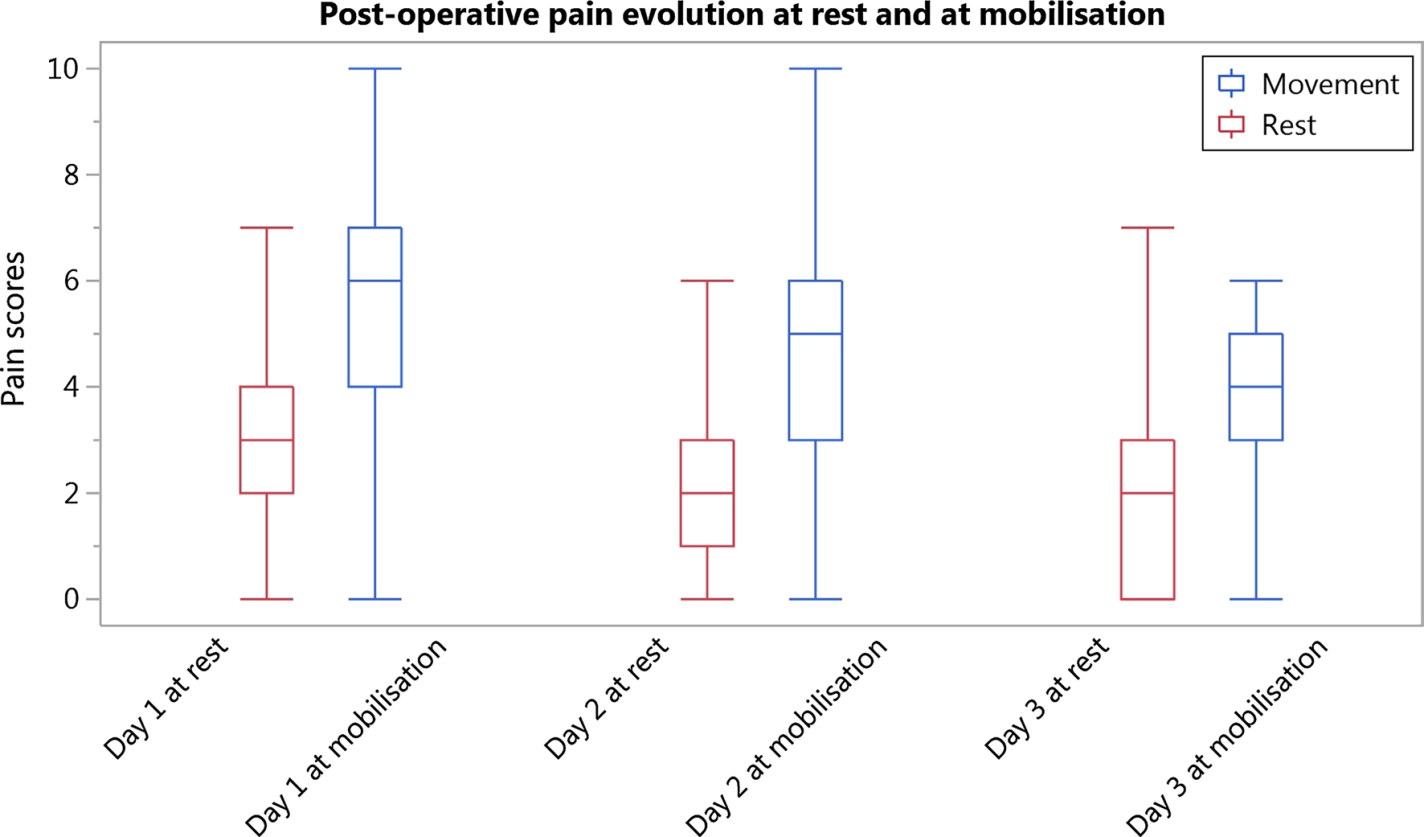
Post-operative pain scores evolution at rest and at mobilisation

Median postoperative pain scores were, at rest and at mobilization respectively 3 [IQR 2–4] and 6 [IQR 4.5–7] on day 1; 2 [IQR 1–3] and 5 [IQR 3–6] on day 2; and 2 [IQR 0–3] and 4 [IQR 3–5] on day 3 (Fig. 1).

Because of inefficacy of the epidural, two donors received a patient-controlled morphine infusion on the day of surgery. Five donors switched to a patient-controlled analgesia with morphine on the next day.

Per Dindo–Clavien classification [72], we counted 30 complications (16 grade 1, 10 grade 2 and 5 grade 3a complications) during the follow-up (Additional file 1). Seventy-five patients did not present any early postoperative complication. Five patients presented more than one complication (a grade 1 and a grade 2 or 3a complication). Two left pneumothorax were due to the insertion of the central venous catheter whereas the third (right) might be secondary to the central venous catheter or the surgery. Median length of stay was 7 days [IQR 6–7].

Two months after the surgery, seven out of the 27 patients (26%) reported persistent pain.

No single element of our protocol was independently associated with any of the postoperative clinical or biological outcome.

## Limitations

We describe here an evidence-based protocol for pain management in living related donor undergoing hepatectomy for liver transplantation. This protocol, based on nine measures, techniques and medications is feasible and has been proposed in a series of hundred living liver donors. Our results show that, even when a protocol based on the highest level of evidence is proposed, the patients’ postoperative scores are comparable to those described in the literature [2, 41]. Furthermore, the number of interventions modulating hyperalgesia precludes any statistical inference in terms of postoperative outcome. To note that psychological factors (e.g. anxiety) were difficult to follow in this context. The language barrier, for example, was an important constraint.

In total, the protocol was entirely applied in only 19 donors. There are several reasons for this. First, each anesthesiologist was free to follow this protocol. Sometimes, due to organizational constraints, the anesthesiologist in charge might not have been one of the core team. A last limitation is the fact that the analysis on pain chronicization suffers from a high rate of loss to follow up.

In conclusion, we describe here an evidence-based protocol for pain management in living related donor undergoing hepatectomy for liver transplantation. This protocol, based on nine measures, techniques and medications is feasible and has been proposed, but not uniformly applied in a series of hundred living liver donors. Taken together, these results show that proposing a protocol based on the most effective therapeutic measures is not sufficient to consider postoperative pain hepatectomy for living donation a resolved issue.

## Additional file

**Additional file 1.** Postoperative complications, graded according to Dindo–Clavien classification in hundred living donors.
