# Supplementary material for: Pain management in living related adult donor hepatectomy: feasibility of an evidence-based protocol in 100 consecutive donors
Source: BMC Res Notes. 2018 Nov 26;11:834. doi: 10.1186/s13104-018-3941-1 (PMC6258399; doi:10.1186/s13104-018-3941-1)
Supplement: Supplementary file 1 — Additional file 1. Postoperative complications, graded according to Dindo–Clavien classification in hundred living donors. [file 13104_2018_3941_MOESM1_ESM.docx]

|  | Complications | Number |
| --- | --- | --- |
| Grade 1 | Small pleural effusion | 3 |
|  | Nausea / vomitings | 7 |
|  | Fever | 3 |
|  | Located haemoperitoneum | 1 |
|  | Transient disorders of haemostasis | 1 |
|  | Keloid scar | 1 |
| Grade 2 | Pulmonary infection | 1 |
|  | Sepsis (streptococcus) | 1 |
|  | Urinary tract infection | 2 |
|  | Corneal erosion | 1 |
|  | Surgical site infection | 1 |
|  | Bowel obstruction | 1 |
|  | Deep venous thrombosis | 1 |
|  | Anaemia (thalassemia) | 1 |
|  | Allergic reactions to iodized contrast agent | 1 |
| Grade 3a | Percutaneously-placed collection drain | 1 |
|  | Pneumothorax (drain/aspiration) | 3 |
|  | Percutaneously-placed drain of biliary leakage | 1 |

**Additional file 1.** Postoperative complications, graded according to Dindo-Clavien classification in hundred living donors.
